# Bioelectrical function and structural assessment of the retina in patients with early stages of Parkinson’s disease (PD)

**DOI:** 10.1007/s10633-015-9503-0

**Published:** 2015-05-14

**Authors:** Barbara Nowacka, Wojciech Lubiński, Krystyna Honczarenko, Andrzej Potemkowski, Krzysztof Safranow

**Affiliations:** Department of Ophthalmology, Pomeranian Medical University, Powstancow Wlkp. 72, 70-111 Szczecin, Poland; Department of Neurology, Pomeranian Medical University, Szczecin, Poland; Department of Clinical Psychology, University of Szczecin, Szczecin, Poland; Department of Biochemistry and Medical Chemistry, Pomeranian Medical University, Szczecin, Poland

**Keywords:** Parkinson’s disease, Retinal bioelectrical function and structure, ERG, OCT

## Abstract

**Purpose:**

To determine bioelectrical function and structural changes of the retina in patients with early stages of Parkinson’s disease (PD).

**Materials and methods:**

Thirty-eight eyes of 20 patients with early idiopathic PD and 38 eyes of 20 healthy age- and sex-matched controls were ophthalmologically examined, including assessment of distance best-corrected visual acuity (DBCVA), slit lamp examination of the anterior and posterior segment of the eye, evaluation of the eye structures: paramacular retinal thickness (RT) and retinal nerve fiber layer (RNFL) thickness with the aid of OCT, and the bioelectrical function by full-field electroretinogram (ERG). Additionally, PD patients were interviewed as to the presence of dopamine-dependent visual functions abnormalities.

**Results:**

In patients with early PD, statistically significant changes in comparison with the control group were observed in ERG. They contained a reduction in mean amplitudes of the scotopic a-wave (rod–cone response), the scotopic oscillatory potentials (OPs)—OP2 and OP3, the photopic b-wave, and a reduction in the overall index (OP1 + OP2 + OP3) and a prolongation of mean peak times of the scotopic OP1, OP2, OP3, OP4 (*p* < 0.05). A questionnaire concerning abnormalities of dopamine-dependent visual functions revealed that PD patients with abnormal peak times of OP1, OP2, and OP3 reported non-specific visual disturbances more frequently in comparison with PD patients with normal peak times of OPs. Other analyzed parameters of ERG, DBCVA, RT, and RNFL did not significantly differ between patients with PD and the control group.

**Conclusion:**

In patients with early PD, bioelectrical dysfunction of the retina was observed in the ERG test, probably as a result of dopamine deficiency in the retina. The results of our study indicate that ERG may also be a useful tool for understanding the reason for non-specific visual disturbances occurring in PD patients.

## Introduction

Parkinson’s disease (PD) is the neurodegenerative disorder characterized by a deficiency of the neurotransmitter—dopamine in the central and peripheral nervous system, including visual pathways. In the eye, dopamine is contained in an A18 subtype of amacrine cells of the retinal inner plexiform layer [1]. Despite of their low density, their widespread dendritic organization and long fine axons ensure overlap with neighboring amacrine cells and bipolar cells and direct influence through synapses [2]. Moreover, every type of retinal neuron may be influenced by dopamine through so-called volume transmission, because it can diffuse over distance of the entire retinal thickness [1]. As dopamine takes part in light adaptation [1, 3], spatial contrast sensitivity and color discrimination [4–6], visuospatial problem solving, spatial working memory, and oculomotor control [6], many PD patients, even in the early stages of the disease, may complain of non-specific visual symptoms. The functional changes may appear even with the normal morphology of the retina and the optic nerve, probably as a result of diminished dopaminergic activity in the visual system. These changes can be detected with the aid of electrophysiological examinations. A few past full-field electroretinogram (ERG) studies reported photopic b-wave amplitude reduction in early PD [7], as well as in patients with different severities of the disease [8–10], but there are also study results that oppose this finding [11–14]. Moreover, Gottlob et al. [10] and Burguera et al. [8] observed reduced amplitudes not only of the scotopic and photopic b-wave, but also of the a-wave. On the other hand, Iudice et al. [15] did not observe any significant differences of scotopic b-wave amplitude of untreated PD patients compared with the controlled group. When the oscillatory potentials (OPs) were studied, Gottlob et al. [10] observed reduced amplitude of second oscillatory potential (OP2), while Kupersmith et al. [16] found no difference between PD and control subjects. Electrophysiological evidence of visual pathology in early PD has also been related to delayed light peak in the electrooculogram (EOG) [7], amplitude reductions in the pattern electroretinogram (PERG) [17], and delays in visually evoked potentials (PVEP) [17].

Optical coherence tomography (OCT) is another method for identifying pathological changes of the retina of PD patients. OCT has been proposed as a useful tool for detecting loss of ganglion cells, secondary to progressive retinal dopaminergic deficiency and amacrine cells’ loss [18]. The paramacular retinal thickness (RT) and the retinal nerve fiber layer (RNFL) thickness near the entry of the optic nerve have been investigated in several studies. However, their results are inconclusive [19–25], and there are no data on anatomical changes exclusively in patients with early PD. Therefore, we decided to determine whether there are any electrophysiological and anatomical changes of the retina in patients with early stages of PD and whether these changes, if present, could at least partially explain nonspecific visual symptoms.

## Methods

### Patients

Thirty-eight eyes of 20 patients aged 60.6 ± 7.9 years with early idiopathic PD (1–1.5 according to Hoehn–Yahr scale, duration of disease under 3 years) and 38 eyes of 20 healthy age- and sex-matched controls (60.9 ± 7.4 years, *p* = 0.75) were enrolled in the study. All participants with any ocular abnormalities of the retina, optic nerve, and ocular media detected via indirect ophthalmoscopy, or previous ocular surgery other than uneventful phacoemulsification, were excluded from the study. Patients with diagnosed early idiopathic PD were referred on ophthalmological examination from the neurological outpatient clinics. The patients’ duration of PD and general medical history were recorded. PD staging was assessed with the modified Hoehn and Yahr (H–Y) scale. Nine of the PD patients were before anti-parkinsonian therapy and had not received any drugs yet. The remainder were under the regimen of anti-parkinsonian treatment. Seven PD patients received precursor of dopamine (l-dopa), and in order to prevent the influence of extraneous dopamine on test results, they were asked to skip their morning dose of anti-parkinsonian treatment prior to the examination. Four PD patients except l-dopa were being treated with biperiden or selegilini hydrochloride, and they were requested to stop the intake of these medications for at least 24 h. All patients enrolled in the present study met these requirements. Patient characteristics are shown in Table 1.

**Table 1:** Characteristics of the study groups and results of the ophthalmological examinations

	PD group	Control group	*p* value
Number of eyes	38	38	ns
Age (years)	60.9 ± 7.7	60.6 ± 7.9	ns
Sex (men/women)	12/8	12/8	ns
PD duration (years)	1.7 ± 1.0	–	–
Stage of PD (H–Y)	1.1 ± 0.2	–	–
DBCVA (log MAR)	0.01 ± 0.02	0.03 ± 0.08	ns
RT (µm)	223.8 ± 14.9	213.8 ± 14.3	ns
RNFL thickness (µm)			
Temporal	63.5 ± 14.4	61.5 ± 12.6	ns
Superior	122.4 ± 17.4	115.2 ± 19.6	ns
Nasal	79.2 ± 18.5	72.0 ± 15.0	ns
Inferior	124.5 ± 17.2	119.3 ± 17.3	ns

Quantitative data are presented as mean ± standard deviation. Qualitative data are presented as number of patients

*PD* Parkinson’s disease, *H–Y* Hoeh and Yahr scale, *DBCVA* distance best-corrected visual acuity, *RT* retinal thickness, *RNFL* *thickness* retinal nerve fiber layer thickness, *ns* not significant (*p* > 0.05)

The study adhered to the tenets of the Declaration of Helsinki. All subjects participating in this study gave their informed written consent. The project was approved by Ethics Committee of the Pomeranian Medical University.

### Procedures

All subjects participating in this study underwent ophthalmological examination of both eyes, including assessment of distance best-corrected visual acuity (DBCVA), slit lamp examination of the anterior and posterior segment of the eye, evaluation of the structure of the macula, the paramacular RT and the peripapillary RNFL thickness in superior, temporal, inferior, and nasal quadrants (fast algorithms, time-domain Stratus OCT, Carl Zeiss Meditec), and ERG (UTAS-E 2000 system, LKC Inc., USA). All parameters despite stimulus strength (1.6 cd s/m^2^ instead of 3.0 cd s/m^2^) were consistent with the current International Society for Clinical Electrophysiology of Vision (ISCEV) Standards [26].

Before recording dark-adapted ERG, pupils were maximally dilated (>6 mm) with 1 % Tropicamidum, and patients were sitting with eyes closed and covered with special black goggles for 30 min. After testing in dark conditions, background light (luminance 32 cd/m^2^) of the Ganzfeld bowl was turned on, and 10 min of light adaptation was performed before recording light-adapted ERGs. The examination was performed with the binocular, full-field (Ganzfeld) stimulation. Two types of electrodes were used: active/reference (right and left)—bipolar contact lens Burian–Allen electrodes and ground—clip gold cup electrode attached to the earlobe. Parameters of the recording system were as follows: amplifiers sensitivity: 10–20–50 µV/div, filters: 0.3–500 Hz (for OPs extraction: 75–500 Hz), notch filters: off, time base: 5 ms/div, and artifact reject threshold: 0 µV. Every response was repeated to study the reproducibility. One of reproducible waveforms was taken for analysis. The flicker response was averaged at 10 sweeps.

## Dark-adapted ERGs

A. Dark-adapted ERG (a dim flash; primarily rod response)—the stimulus was a dim white flash of 0.012 cd s/m^2^; analyzed parameters: amplitude and peak time of the b-wave.
B. Dark-adapted ERG (a strong flash; rod–cone response)—the stimulus was a white flash of 1.6 cd s/m^2^; analyzed parameters: amplitude and peak time of the a- and b-waves.
C. Dark-adapted oscillatory potentials—1.6 cd s/m^2^ flash stimulation; the second waveform was retained; analyzed parameters: amplitude and peak time of the first four oscillatory waves (OP1, OP2, OP3, OP4) and the overall index (a sum of OP1 + OP2 + OP3 amplitudes) [26].

## Light-adapted ERGs

A. Light-adapted ERG (primarily cone response)—the stimulus was a white flash of 1.6 cd s/m^2^; analyzed parameters: the amplitude and peak time of the a- and b-waves.
B. Light-adapted flicker ERG (cone response)—flickering 1.6 cd s/m^2^ flashes presented at a rate of 30 stimuli per second (30 Hz); during the first 5 s of pre-adaptation waveforms were discarded in order to reach stable conditions; analyzed parameters: the peak-to-though amplitude and peak timing from the midpoint of the stimulus flash to the following peak, which was calculated automatically from 10 averaged recordings.

Additionally, PD patients were interviewed as to the presence of dopamine-dependent visual function abnormalities: difficulties in light adaptation and smooth pursuit, decreased contrast sensitivity, and abnormalities in color vision.

### Statistical analysis

Since distributions of most analyzed quantitative variables were significantly different than normal distribution (*p* < 0.05, Shapiro–Wilk test), the nonparametric Mann–Whitney *U* test was used for comparisons between groups. A *p* value < 0.05 was considered significant. To address the problem of multiple comparisons, false discovery rate (FDR) methodology was used [27]. *Q* values indicating the expected proportion of incorrectly rejected null hypotheses (“false discoveries”) were calculated for all comparisons. The *q* value of an individual hypothesis test is the minimum FDR at which the test may be called significant.

In analyzing individual patients’ results, the electrophysiological parameters were considered as normal if they were between 2.5 and 97.5 percentile.

## Results

The distance best-corrected visual acuity was good in all subjects participating to the study and did not significantly differ between PD patients and controls. Retinal and RNFL thicknesses’ differences were also statistically insignificant. The structure of the macula was normal in all patients. Results of the above examinations are summarized in Table 1.

**Fig. 1:**
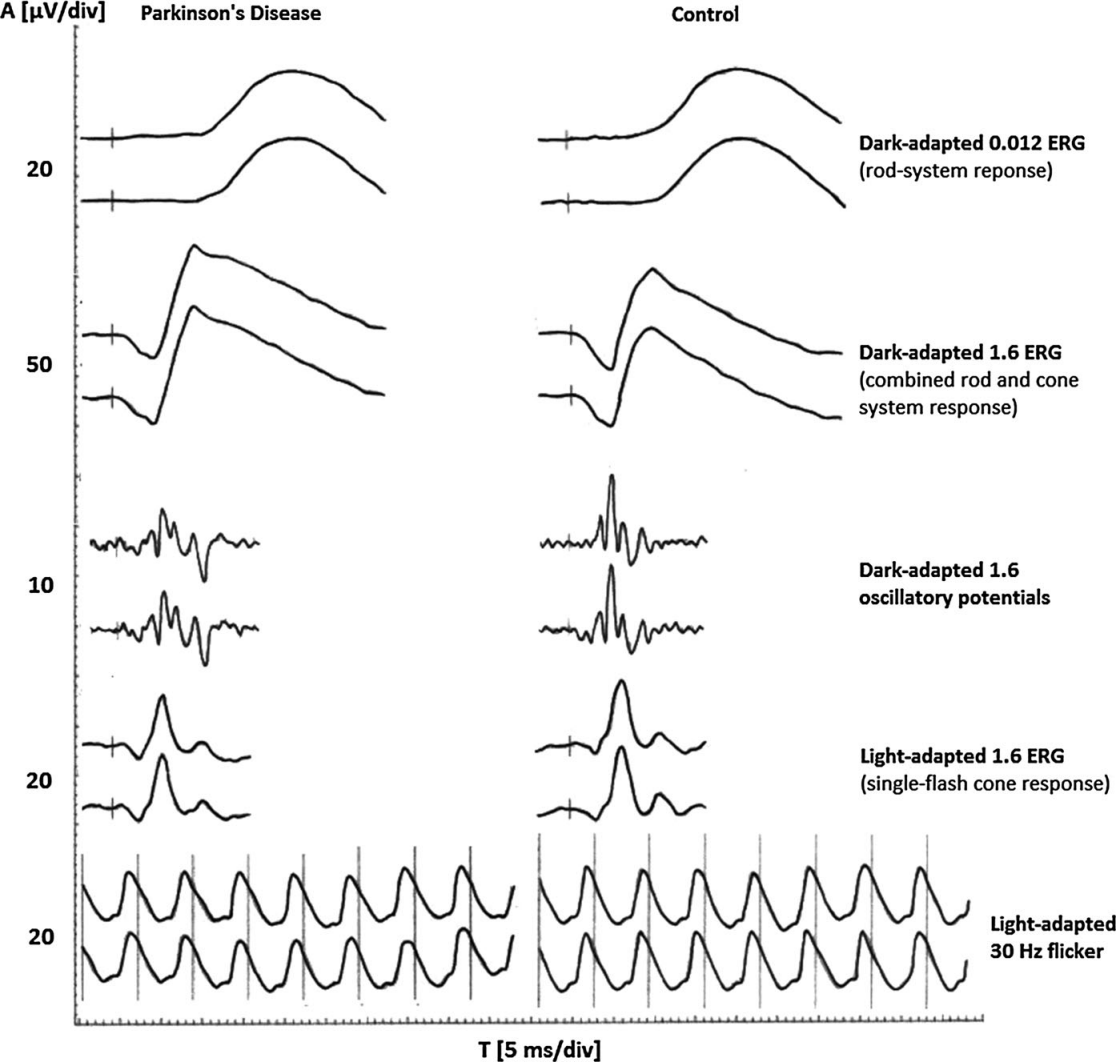
Example of reduced amplitudes of the scotopic a-wave and the photopic b-wave, and the abnormal OPs obtained from the eye of a PD patient in comparison with the normal results of a control case

Statistically significant ERG differences between patients with early stages of PD and control subjects were observed. They contained a reduction in mean amplitudes of the scotopic a-wave (rod–cone response), the scotopic oscillatory potentials (OPs)—OP2 and OP3, the photopic b-wave, and reduction in the overall index (OP1 + OP2 + OP3) and a prolongation of mean peak times of the scotopic OP1, OP2, OP3, and OP4 (*p* < 0.05). It is worth noting that the scotopic a-wave peak time was at the borderline of statistical significance (*p* = 0.07). Other analyzed parameters of ERG recordings did not significantly differ between patients with PD and the control group. After FDR correction for multiple comparisons significance (*q* value < 0.05) was obtained for three differences: scotopic a-wave amplitude, scotopic OP1 and OP2 peak times. These differences have the lowest risk of being false positive. Five additional differences (scotopic OP3 and OP4 peak times, OP3 amplitude, the overall index, and photopic b-wave amplitude) were of borderline significance with *q* value between 0.05 and 0.1. It means that among the eight differences with *q* value < 0.1, only one of ten (i.e. one difference) is expected to be found a false positive. The total number of comparisons was 27. Results of ERG test are summarized in Table 2. The example of reduced amplitudes of the scotopic a-wave and the photopic b-wave, and the abnormal OPs obtained from the eye of a PD patient in comparison with the normal results of a control case is shown in Fig. 1.

**Table 2:** Comparison of ERG results from 38 eyes of 20 patients with early stages of PD and controls

	Wave	Group	N	M ± SD	Min	Med	Max
Dark-adapted ERG (dim flash)	b-wave A (µV)	PD C	+ −	113.6 ± 44.5 134.7 ± 75.3	43.4 41.3	110.3 119.4	210.9 408.0
	b-wave PT (ms)	PD C	+ +	119.7 ± 10.0 120.7 ± 7.3	105.0 102.0	119.0 121.0	132.5 140.0
Dark-adapted ERG (strong flash)	a-wave A (µV)	PD C	+ +	**137.5** **±** 52.2*** ^**#**^ **179.9** **±** 14.2*** ^**#**^	**11.5** **58.1**	**144.3** **182.4**	**237.0** **292.5**
	a-wave PT (ms)	PD C	− +	24.1 ± 1.9 23.8 ± 0.9	17.5 22.0	24.6 23.8	29.0 26.5
	b-wave A (µV)	PD C	+ −	428.8 ± 100.7 431.9 ± 101.9	212.4 261.2	417.4 414.6	611.4 685.3
	b-wave PT (ms)	PD C	− −	48.1 ± 3.7 48.4 ± 3.2	44.0 42.0	47.5 47.3	60.5 53.5
Dark-adapted ERG oscillatory potentials	OP1						
	A (µV)	PD C	+ +	28.2 ± 11.3 31.8 ± 13.6	8.3 10.3	29.6 29.0	48.7 58.8
	PT (ms)	PD C	− +	**21.5** **±** 3.5*** ^#^ **19.2** **±** 1.3*** ^#^	**18.5** **16.5**	**20.0** **19.0**	**31.0** **22.0**
	OP2						
	A (µV)	PD C	+ +	**43.9** **±** 19.2* **56.2** **±** 25.2*	**10.7** **17.6**	**44.4** **57.6**	**83.8** **114.6**
	PT (ms)	PD C	− −	**28.5** **±** 3.2** ^#^ **26.6** **±** 1.3** ^#^	**25.5** **24.5**	**27.0** **26.5**	**37.0** **29.5**
	OP3						
	A (µV)	PD C	+ +	**34.5** **±** **14.9** ^*^^ **43.9** **±** **16.5** ^*^^	**7.2** **15.9**	**33.8** **41.1**	**75.7** **76.4**
	PT (ms)	PD C	− −	**35.2** **±** **3.6** ^**^^ **33.4** **±** **1.8** ^**^^	**31.0** **30.0**	**34.0** **33.0**	**44.5** **39.5**
	OP4						
	A (µV)	PD C	− −	28.9 ± 19.9 23.5 ± 15.1	2.2 3.2	21.9 20.1	73.0 70.9
	PT (ms)	PD C	+ −	**43.9** **±** **3.4** ^*^^ **42.3** **±** **3.6** ^*^^	**39.0** **37.0**	**43.3** **41.5**	**53.5** **55.0**
	Overall index (µV)	PD C	+ +	**106.7** **±** **36.9** ^*^^ **131.9** **±** **47.0** ^*^^	**50.0** **53.7**	**107.9** **130.5**	**192.7** **233.5**
Light-adapted ERG	a-wave A (µV)	PD C	+ +	32.1 ± 22.7 27.1 ± 14.2	4.8 1.1	30.6 26.2	88.2 66.1
	a-wave PT (ms)	PD C	− +	15.7 ± 1.5 15.5 ± 1.0	11.0 13.0	15.5 15.5	19.0 18.0
	b-wave A (µV)	PD C	+ +	**85.9** **±** **35.1** ^*^^ **107.8** **±** **43.0** ^*^^	**29.5** **42.6**	**88.9** **103.4**	**160.5** **229.4**
	b-wave PT (ms)	PD C	− −	30.8 ± 2.2 30.6 ± 1.3	28.5 28.0	29.5 30.8	41.5 32.5
Light-adapted flicker ERG	A (µV)	PD C	+ +	59.9 ± 20.9 68.2 ± 24.1	27.4 26.3	61.5 65.3	97.0 120.0
	PT (ms)	PD C	− +	31.6 ± 4.0 30.5 ± 1.6	27.6 27.8	31.0 30.3	34.8 35.5

*A* amplitude, *PT* peak time, *PD* Parkinson’s disease, *C* control, *N* normal distribution, *M* *±* *SD* mean ± standard deviation, *Min* minimal value, *Med* median, *Max* maximum value, *Overall index* OP1 + OP2 + OP3

Statistically significant (bold): * *p* < 0.05; ** *p* < 0.01; *** *p* < 0.001

^#^Statistically significant (*q* < 0.05) after FDR correction for multiple comparisons

^^^Borderline statistical significance (*q* < 0.1) after FDR correction for multiple comparisons

When each of the 38 examined eyes of 20 PD patients was analyzed separately, the results from eight (21 %) eyes of six PD patients deviated from the normal values for OPs peak times. On this basis, all 20 PD patients were divided into two groups: six patients with abnormal OPs and 14 patients with normal OPs. The patients with abnormal OPs more frequently reported non-specific visual disturbances: difficulties in light adaptation (3/6 vs. 2/14 patients with abnormal OPs vs. normal OPs, respectively) and smooth pursuit (1/6 vs. 0/14 patients), decreased contrast sensitivity (3/6 vs. 1/14 patients), abnormalities of color vision (1/6 vs. 0/14 patients). The results of the comparison of the frequency of dopamine-dependent visual function abnormalities and the results of ERG—OPs in PD patients are summarized in Table 3.

**Table 3:** Comparison of the frequency of dopamine-dependent visual function abnormalities and the results of ERG—oscillatory potentials in 20 PD patients

Visual symptoms	ERG—OPs	
	↑ PT (*n* = 6/20)	normal PT (*n* = 14/20)
Difficulties in light adaptation	3/6 (50 %)	2/14 (14.3 %)
Decreased contrast sensitivity	3/6 (50 %)	1/14 (7.1 %)
Abnormalities of color vision	1/6 (16.7 %)	0/14 (0 %)
Difficulties in smooth pursuit	1/6 (16.7 %)	0/14 (0 %)

Data are presented as number of patients and percentage

*PD* Parkinson’s disease, *PT* peak time, *OPs* oscillatory potentials, *n* number of patients

## Discussion

In this study, it was shown for the first time, that there were no changes in RT and RNFL thickness in patients with early stages of PD. The results of previous OCT studies in patients with more advanced stages of PD were inconclusive. Some of these studies using time-domain OCT [19, 20] pointed at a decreased RNFL thickness in the inferior and temporal quadrant. However, some other studies revealed no difference in the inferior, superior, nasal, or temporal sectors between PD and control cases [21, 22]. Inzelberd et al. [19] suggested that loss of RNFL thickness in PD might be a result of reduced dopaminergic input to a subset of ganglion cells, which may cause atrophy, and localized thinning of RNFL. Also, results of OCT studies concerning RT measurements in PD patients are inconclusive. Consistent with our results, Archibald et al. [22] recently showed no significant RT changes with time-domain OCT, but significant differences in macular thickness were detected with spectral-domain OCT [23, 24]. However, there is also a study utilizing the same methodology that opposes this finding [25].

The results of our study indicate that even with the absence of structural changes in the retina, there might be electrophysiologically detectable dysfunctions in the retinae of patients with early stages of PD. We observed a reduction in mean amplitudes of the scotopic a-wave (rod-cone response), the scotopic oscillatory potentials (OPs)—OP2 and OP3, the photopic b-wave, and a reduction in the overall index (OP1 + OP2 + OP3) and a prolongation of mean peak times of the scotopic OP1, OP2, OP3, and OP4 (*p* < 0.05). In the literature, only a few reports can be found of studies describing ERG recordings in PD patients in general (advancement of PD ranged from 1 to 4 according to Hoehn–Yahr scale). Moreover, patients were examined in the course of their treatment with anti-parkinsonian drugs (including l-dopa), which might have influenced ERG results. It is known from a study by Jaffe et al. [28] that exogenous dopamine enhanced the photopic b-wave almost by one-fourth. Moreover, in some studies, the experimental group consisted of patients with parkinsonism of other than idiopathic etiologies (for example arteriosclerotic) [12–14]. In the course of PD, the reduced amplitudes of the scotopic and photopic a- and b-waves were observed [8–10]. However, there are also studies which did not confirm these findings [11–14]. When OPs were analyzed, no difference in OPs peak times were found [10, 16]. However, the reduced amplitude of the photopic OP2 was observed [10].

According to our best knowledge, there is only one past study concerning ERG changes in untreated patients with stage I of PD according to the Hoehn–Yahr scale. Despite some differences in the methodology (e.g., stimulation of red flash), Ikeda et al. [7] observed significant changes in b-wave amplitude in early PD patients compared with controls, which is consistent with our results. Our findings are also consistent with results of Iudice et al [15] who did not observe any significant differences in the scotopic b-wave amplitude of untreated PD patients compared with controls, but the degree of advancement of PD was not specified.

In this study, we observed reduced amplitude of scotopic a-wave (rod-cone response) in PD group. Moreover, when we carefully analyzed collected data of scotopic a-wave, we observed that 79 % of eyes of the controls achieved the amplitude of more 165 µV, whereas only 29 % eyes of PD patients showed these results. The difference was statistically significant (Mann–Whitney *U* test, *p* = 0.00018). The results of animal studies indicate that a-wave (more precisely the fast P-III component of the ERG) mostly reflects light-induced activity of the photoreceptors [29]. Meanwhile, photoreceptors seems to be a subject of a number of dopamine-mediated mechanisms. Dopamine, acting through a D2 receptor, modulate the voltage-gated calcium current [30], a hyperpolarization-dependent current [31], and coupling between rods and cones [32]. Moreover, Shulman and Fox [33] report that activation of the D4 receptor inhibits the Na/K ATPase of rat rods. In relation to considerations of dopamine receptors, their number and/or sensitivity may be up-regulated when dopamine concentrations are very low [34]. The retinal dopamine seems to be a primary factor coordinating shift from nighttime to daytime vision, thus functional transition from a rod- to cone-dominated state [35, 36]. Therefore, it seems sensible to hypothesize that lower concentration of retinal dopamine in course of PD may cause disruption of one or more dopamine-mediated mechanisms in the photoreceptors, what was observed as reduction in the scotopic a-wave amplitude in the present study. We also showed increased peak times and reduced amplitudes of the OPs. On the basis of results of previous studies, OPs are thought to reflect neural interactions between amacrine, ganglion, and bipolar cells, and the ON pathways appear to play a critical role in OPs generation [37–40]. Meanwhile, dopaminergic amacrine cells A18 are definitely involved in ON pathways [41]. Results of animal studies demonstrated that despite the anatomical projections of A18 cells in the off sublamina of the inner retina, no OFF responses were recorded in dopaminergic amacrine cells [41]. Moreover, results of animal studies using reserpine—an indole alkaloid causing depletion of monoamine neurotransmitters (dopamine, norepinephrine, serotonin) in the synapses—showed absence of OPs [42, 43]. Oscillatory potentials reappeared when l-dopa was injected intravitreally. Thus, it is reasonable to suppose that dopaminergic amacrine cells A18 make a contribution to OPs generation. We also observed reduction in photopic b-wave. The results of animal studies indicate that cellular origin of b-wave are mostly the ON bipolar cells [44–46]. The results of other animal studies on the contribution to the shape of the ERG b-wave by third-order retinal neurons indicate that amacrine cells might modulate its kinetics and amplitude [47]. Moreover, as dopamine takes part in light adaptation [1, 3], it may be assumed that impairment of this process due to decreased dopamine concentration in the PD retinas might cause photopic b-wave amplitude reduction. In a present study, PD patients also achieved worse results of amplitude and peak time of flicker ERG than controls, but the difference was not statistically significant. We assume that if the examined sample was larger, the difference could achieve statistical significance. Although the complexity of dopamine function at multiple levels in the outer and inner retina in producing alterations to the flow of visual information, it can be supposed that decreased dopamine concentration in PD may be a cause of presented changes in ERG.

The analysis of individual PD patients’ ERG results revealed that patients with abnormal OPs more frequently reported dopamine-dependent visual disturbances, such as difficulties in light adaptation and decreased contrast sensitivity in comparison with individuals in the subgroup with normal OPs peak times. The results of the previous studies indicate that visual dysfunction in the course of PD appeared to be due to retinal dopaminergic deficiency and impairment of central visual system [48]. Our results suggest that in the eyes of PD patients with dopamine-dependent visual function abnormalities, this dopaminergic retinal defect is present. Especially, that electrical activity of the A18 cells is associated with dopamine release from dopaminergic neurons in the brain [49, 50]. Further research is needed to determine if higher doses of exogenous dopamine reduces the occurrence of these visual disturbances.

## Conclusion

The result of this study confirms previous findings [7–10] that there is a dopaminergic defect of the PD patients’ retina, and it could be detected by ERG. However, we expanded our knowledge in that even in patients with early PD, there is bioelectrical dysfunction of the retina, and it is not only manifested by b-wave reduction as Ikeda et al. [7] observed, but also by reduction in scotopic a-wave and OPs amplitudes and prolongation of scotopic OPs peak times. The ERG may be also considered as a useful tool for understanding the reason of non-specific visual disturbances occurring in PD patients. However, further research is needed to confirm our findings.
